# miRNA profile at diagnosis predicts treatment outcome in patients with B-chronic lymphocytic leukemia: A FILO study

**DOI:** 10.3389/fimmu.2022.983771

**Published:** 2022-10-17

**Authors:** Isabelle Duroux-Richard, Anne-Laure Gagez, Elina Alaterre, Rémi Letestu, Olfa Khalifa, Christian Jorgensen, Stéphane Leprêtre, Emmanuelle Tchernonog, Jérôme Moreaux, Guillaume Cartron, Florence Apparailly

**Affiliations:** ^1^ Institute of Regenerative Medicine and Biotherapy, INSERM, U1183, University of Montpellier, Montpellier, France; ^2^ Department of Clinical Hematology, University Hospital Montpellier, Montpellier, France; ^3^ Institute of Human Genetics (IGH), CNRS, University of Montpellier, Montpellier, France; ^4^ Department of Biological Hematology, APHP, Groupe hospitalier hôpitaux universitaires Paris Seine Saint Denis (GH HUPSSD), Hospital Avicenne, Bobigny, France; ^5^ Clinical Department for osteoarticular diseases, University hospital Lapeyronie, Montpellier, France; ^6^ Department of Hematology, INSERM, U1245, Centre Henri Becquerel, Normandie Univ UNIROUEN, Rouen, France; ^7^ Department of Biological Hematology, Laboratory for Monitoring Innovative Therapies, University Hospital Montpellier, Montpellier, France; ^8^ Institut Universitaire de France (IUF), Paris, France; ^9^ CNRS UMR 5535, University of Montpellier, Montpellier, France

**Keywords:** microRNA, prediction, treatment outcome, diagnosis, B-CLL, trancriptomics

## Abstract

During many years, chemo-immunotherapy fludarabine-cyclophosphamide-rituximab (FCR) was the gold standard for first line treatment of medically fit patients with symptomatic B-chronic lymphocytic leukemia (CLL). Over the last decade, targeted biotherapies have revolutionized the treatment of B-CLL patients and almost entirely supplanted FCR. However, no biomarker still exists to predict the complete remission (CR) with undetectable minimal residual disease (uMRD) in bone marrow (BM), which remains the best predictive factor for survival. MicroRNAs represent a class of molecular biomarkers which expression is altered in B-CLL. Our study aimed at identifying before treatment blood miRNAs that predict treatment outcome in previously untreated B-CLL patients (NCT 01370772, https://clinicaltrials.gov/ct2/show/NCT01370772). Using hierarchical clustering of miRNA expression profiles discriminating 8 patients who achieved CR with BM uMRD from 8 patients who did not achieve CR and displayed detectable BM MRD, we identified 25 miRNAs differentially expressed before treatment. The expression of 11 miRNAs was further validated on a larger cohort (n=123). Based on the dosage of 5 miRNAs at diagnosis, a decision tree was constructed to predict treatment outcome. We identified 6 groups of patients with a distinct probability of being CR with BM uMRD to FCR treatment, ranging from 72% (miR-125b, miR-15b and miR-181c high) to 4% (miR-125b and miR-193b low). None of the patients displaying high expression levels of miR-125b, miR-15b and miR-181c relapsed during study follow-up. In contrast, patients with low miR-15b and high miR-412, or with low miR-125b and miR-193b, demonstrated significant low PFS. RNA sequencing of blood at diagnosis identified that patients relapsing after treatment are characterized by significant enrichment of gene signatures related to cell cycle, *MYC* target genes, metabolism and translation regulation. Conversely, patients achieving CR with BM uMRD displayed significant enrichment in genes related to communication between CLL cells and the microenvironment, immune system activation and upregulation of polycomb PRC2 complex target genes. Our results suggest that blood miRNAs are potent predictive biomarkers for FCR treatment efficacy and might be implicated in the FCR efficacy in B-CLL patients, providing new insight into unmet need for the treatment of B-CLL patients and identifying pathways predictive of patients’ remission.

**Clinical trial registration:**

ClinicalTrials.gov, identifier NCT 01370772.

## Introduction

B-cell chronic lymphocytic leukemia (CLL) is the most common adult leukemia in western countries. The median age of patients at diagnosis is 65 years, with only 10 to 15% under 50 years of age (1). The prognostic of CLL has been largely improved by immune-chemotherapy associating an anti-CD20 monoclonal antibody (rituximab) with chemotherapy. A German study group has demonstrated that the association of fludarabine, cyclophosphamide and rituximab (FCR) improved significantly progression-free and overall survivals of patients fewer than 65 years (2). Similar results were also demonstrated with chlorambucil and obinutuzumab, a glycomodified anti-CD20 monoclonal antibody for older unfit patients (3, 4). Immuno-chemotherapy induces significant hematologic and infectious toxicities but the goal is then to obtain the best control of disease with complete response (CR) and undetectable minimal residual disease (uMRD). Over the last decade, small molecule inhibitors of the B-cell receptor (BTKi, PI3Ki) and B-cell leukemia/lymphoma 2 (BCL-2i) signaling pathways (ibrutinib, idelalisib, venetoclax) have revolutionized therapeutic options for B-CLL patients, becoming particularly frontline therapies in high-risk patients. BTK inhibitors however mostly provide partial response (PR) with rare uMRD, thus requiring long-term continuous therapy to achieve durable disease control (5, 6) that is associated with a significant financial burden (7). Finally, although B-CLL is generally an indolent malignancy, it presents a wide clinical heterogeneity with highly variable clinical course and a significant number of patients show aggressive clinical course with treatment resistance or relapse. Despite huge progress in shifting from chemotherapy to chemotherapy-free regimens, powerful tools for outcome prediction are still missing to optimally guide therapeutic decisions.

Many prognostic factors have been described for CLL patients (8). They can be related to the patient, the disease characteristics or the treatment response. Age, genetic parameters and comorbidities are probably the most important prognostic factors related to the patient himself that limit the physician in the choice of an appropriate treatment. Among the numerous prognostic factors related to the disease, the (un)mutated status of the immunoglobulin heavy chain variable region (*IGHV*) and unfavorable genetic abnormality (17p deletion or *TP53* mutations) are very powerful, this latter one being clearly associated with a poor probability of response to chemotherapy and survival (9). Inferior response duration and survival have been revealed to be associated with the quality of response to treatment, irrespective of the treatment used. Thus, achieving uMRD after treatment is associated with superior progression-free survival (PFS) and overall survival (OS) (10). MRD status is the single and the best post-treatment predictive factor of long-term outcome after treatment and therefore has become the goal of immuno-chemotherapy (11, 12). Although it is generally admitted that bone marrow (BM) uMRD is more sensitive for outcome prediction, peripheral blood is often chosen by physician because of easier access and comfort for patient (13). The probability to obtain BM uMRD is however different according to treatment, the best probability being obtained with FCR regimen (44%), whereas chlorambucil-obinutuzumab allowed lower rate of uMRD (19.5%) (11, 12, 14). MD Anderson Cancer center reported long-term results of a cohort of patients receiving FCR, demonstrating that mutated CLL patients with uMRD after FCR reached very long PFS without any relapse after 7 years of follow-up, arguing for the continued use of chemo-immunotherapy in this patient’s subgroup (14). However, the probability to obtain uMRD for mutated CLL patients was reported to be 51%, highlighting the interest to predict the probability to obtain uMRD to expose to chemotherapy only patients with high probability to have uMRD at the end of immune-chemotherapy.

MicroRNAs (miRNAs) are a class of small non-coding RNAs that regulate gene expression at the post-transcriptional level. Several studies correlated miRNAs with clinical characteristics or outcome of B-CLL patients, leading to the identification of B-CLL subgroups with worst outcome (15). Patients’ refractory to fludarabine exhibited significantly higher expression levels of miR-21, miR-148a and miR-222 than fludarabine-sensitive patients (16). Activation of the TP53-reponsive genes was only found in fludarabine responsive patients, suggesting a possible link between abnormal miRNA expression and TP53 dysfunctional pathway in non-responder patients. Many publications have reported significant levels of miRNAs in serum and other body fluids in physiological and pathological conditions, raising the possibility that miRNAs may be probed in the circulation and serve as diagnostic or prognostic outcome biomarkers (17–19). Plasma miRNAs can discriminate CLL samples from controls (20) and complete remission is associated with low levels of miR-155 before treatment (21). We demonstrated that miRNA might predict lymphodepletion with rituximab alone in untreated CLL patients (22). Visone et al. found that blood expression levels of miR-181b decreased in progressive B-CLL patients but not in patients with a stable disease (23). However, no miRNA-based predictive model of treatment outcome has been proposed yet.

In this context, the present study aimed at evaluating miRNA blood transcriptome at diagnosis in previously untreated active B-CLL to identify miRNAs that predict patients with CR who will reach BM uMRD 3 months after immuno-chemotherapy by FCR.

## Materials and methods

### Patients

A prospective, randomized, open-label, phase II study (CLL2010FMP, NCT 01370772) has included 140 treatment-naive patients (aged 18-65 years) diagnosed with confirmed chronic lymphocytic leukemia according to IWCLL 2008 criteria and Binet stage C or with active Binet stage A or B (24). An additional inclusion criterion was the absence of 17p deletion, assessed by FISH (<10% positive nuclei). Patients were stratified according to *IGHV* mutational status, FISH analysis (11q deletion or not) and were randomly assigned to receive either 6 cycles of chemo-immunotherapy FCR every 28 days or Dense-FCR with an intensified rituximab pre-phase (6500 mg total dose) before the standard treatment FCR, as previously published (25). Patients were assessed 9 months (M9) after the first course of FCR in the two arms and at least 3 months after the end of the treatment whatever the number of the cycle. All CT-scan were centrally reviewed for response assessment. According to IWCLL 2008 guidelines, complete remission patients group include complete remission with incomplete marrow recovery patients (24).

### Cell surface CD20 expression analysis

CD20 expression was quantified using CD20-PE QuantiBRITE™ reagents (Ratio 1:1) according to manufacturer’s recommendations (BD Biosciences, Le Pont-de-Claix, France). Qualibration and quantification were performed using a FACSCANTO II cytometer (BD Biosciences, Le Pont-de-Claix, France).

### Minimal residual disease determination

An 8-color combination comprising CD19, CD20, CD5, CD43, CD79b, CD81, CD22, CD45 and CD200 was performed by flow cytometry (FACSCANTO II cytometer, BD Biosciences, Le Pont-de-Claix, France) to evaluate the immunophenotypic response of B-CLL patients in both blood and bone marrow (BM) at M9, as previously described (12). Presence of MRD was defined as the detection of one or more CLL cell per 10 000 leukocytes (24).

### miRNA transcriptomics and validation

Blood was collected before treatment in PAXgene and total RNAs, including small RNAs, were extracted as previously described (22). RNA quality was assessed using the 2100 Bioanalyzer assay (Agilent, Les Ulis, France), and according to the criteria of the Minimum Information for publication of Quantitative real-time PCR Experiments MIQE guidelines, only samples with a RIN>8 were used. Sixteen patients were analyzed with the TaqMan Low-Density Array (TLDA) technology as previously described (22) and divided in two groups according to treatment response outcome. One group was composed of 8 patients with CR with blood and BM uMRD. The other group was composed of 8 patients with no-CR and detectable MRD. Validation of miRNA expression levels were quantified in a second cohort of 100 treatment-naive patients using multiplex Taqman microRNA assays (Life Technologies, Saint Aubin, France) as previously described (22).

### Gene expression profiling

Gene expression profiles (GEP) were obtained using RNA sequencing (RNA-seq). The RNA-seq library preparation was completed with 150 ng of input RNA using the Illumina TrueSeq Stranded mRNA Library Prep Kit. Pairedend RNA-seq was performed with an Illumina NextSeq sequencing instrument (Helixio, Clermont-Ferrand, France). RNA-seq read pairs were mapped to the reference human GRCh37 genome using the STAR aligner (26). All of the statistical analyses were performed with the R statistics software (version 3.2.3; available from https://www.r-project.org) and with R packages developed by the BioConductor project (available from https://www.bioconductor.org/) (27). The expression level of each gene was summarized and normalized using the DESeq2 R/Bioconductor package (28). Differential expression analysis was performed using the DESeq2 pipeline (28). p-values were adjusted to control the global FDR across all comparisons with the default option of the DESeq2 package.

### Statistical analyses

Distributions of data were tested with the Shapiro-Wilk test. X^2^ or Fisher test was used for categorical data. For numerical data, comparisons of medians were performed using Student T or Mann-Whitney test. Spearman’s correlation test was used to assess the association between two numerical data. Multivariate analysis was performed using logistic regression by backward selection using Student T test (p<0.05 as significant model). To establish the decisional tree, the receiver operating characteristics (ROC) of miRNA was constructed to determine the threshold able to predict complete response with blood and BM uMRD associated with the best sensitivity and specificity according to the Youden index (29). A probability p<0.05 was considered statistically significant. Progression Free Survival (PFS) was measured from the date of the initiation of the treatment to the date of relapse and/or progression as a result of B-CLL or acute treatment toxicity. PFS analysis was calculated using the Kaplan-Meier method and comparisons made using the log-rank test. Landmark analyses were performed from the time of MRD assessment (3 months after the end of the treatment) to assess the impact of treatment outcome (including MRD) on PFS. Hazard ratios, including 95% confidence interval in univariate analysis and multivariate analysis were performed using Cox regression. All statistical analyses were performed at the conventional two-tailed α level of 0.05 using R software version 3.0.2.10.

## Results

### Patients’ characteristics

Among the 140 patients recruited in the CLL2010FMP study, pre-treatment blood samples of only 123 patients were available for RNA analyses. Patients’ characteristics are presented in **Table 1**. Median age was 58.5 years (interquartile range (IQR): 52.8-61.8), 26.8% were women and 74% were Binet stage A or B. Cytogenetic analyses identified del(13q), del(11q) and trisomy 12 in 56, 20 and 11% of B-CLL patients. The median lymphocyte count before treatment was 71 G/L (IQR: 29.6-115.3). Among 123 patients for whom pre-treatment RNA samples were available for analysis, 5 did not reach quality control criteria. Nine months after FCR treatment, 65 patients (52.85%) were in complete remission (CR) and 53 patients (43.09%) were not in complete remission (no-CR). There was no statistical difference in the CR versus no-CR between treatment arms as previously published (25). Blood MRD was undetectable in 69 out of the 108 samples analyzed (63.89%), with similar distribution in both treatment arms (35 in the FCR arm and 34 in the Dense-FCR arm). MRD in BM was undetectable in 40 out of the 95 patients analyzed (42.11%), with again comparable distribution between both arms (21 in the FCR arm and 19 in the Dense-FCR arm). When combining CR with BM uMRD, 27 out of 107 patients (25.23%) were responders to the treatment, 24.53% and 25.93% distributed in the FCR arm and Dense-FCR arms, respectively. Finally, 80 patients (74.77%) equally divided in both FCR and Dense-FCR arms (64.52% and 65.57%, respectively) were no-responders. Overall response rate for the entire cohort was 94% including 53% of CR or Cri (25). Associations between pre-treatment characteristics, CR, peripheral blood (PB), BM, and CR with PB and BM uMRD rates are shown in **Supplemental Table 1**. Univariate analysis showed significant differences in CR rate according to age, Binet stage, β2-microglobulin or number of cycles received (p=0.026, p<0.0001, p=0.003 and p<0.001, respectively). There was no significant difference in CR rate according to *IGHV* mutational status or cytogenetic abnormalities. Only trisomy 12 was statistically significant for PB uMRD rate (p=0.021), but 34% (71/108) of trisomy 12 data were missed. For BM uMRD rate, only lymphocytes count at D0 was significant (p=0.020). Patients who were in Binet stage AB, had trisomy 12, low lymphocytes count at D0 and completed 6 cycles of therapy were more likely to achieve CR with PB and BM uMRD (p=0.006, p=0.026, p=0.004, p=0.038, respectively).

**Table 1:** Patients’ characteristics.

	Cohort (n = 123)	
	n (%)	Median (IQR)
**Age (years)**	–	58.45 (52.82-61-83)
**Women**	33 (26.83)	–
**Binet stage AB**	91 (73.98)	–
**ECOG 0**	86 (69.92)	–
**Unmutated *IGHV***	75/119 (63.03)	–
**Cytogenetic abnormalities**		
**Del (13q)**	54/96 (56.25)	–
**Del (11q)**	24/120 (20.00)	–
**Trisomy 12**	9/82 (10.98)	
**Lymphocyte count (G/L)**	–	71.00 (29.62-115.25)
**β2 microglabulin (mg/L)**	114 (92.68)	3.05 (2.35-4.00)
**IL-10 competent B cells**	44 (35.77)	2.97 (0.98—9.58)
***FCGR3A***	115 (93.50)	–
**V/V**	14 (12.17)	–
**V/F**	55 (47.83)	–
**F/F**	46 (40.00)	–
**Treatment arm FCR**	62 (50.41)	–

IQR, interquartile range.

### miRNAs associated with complete remission with bone marrow undetectable minimum residual disease

**Figure 1:**
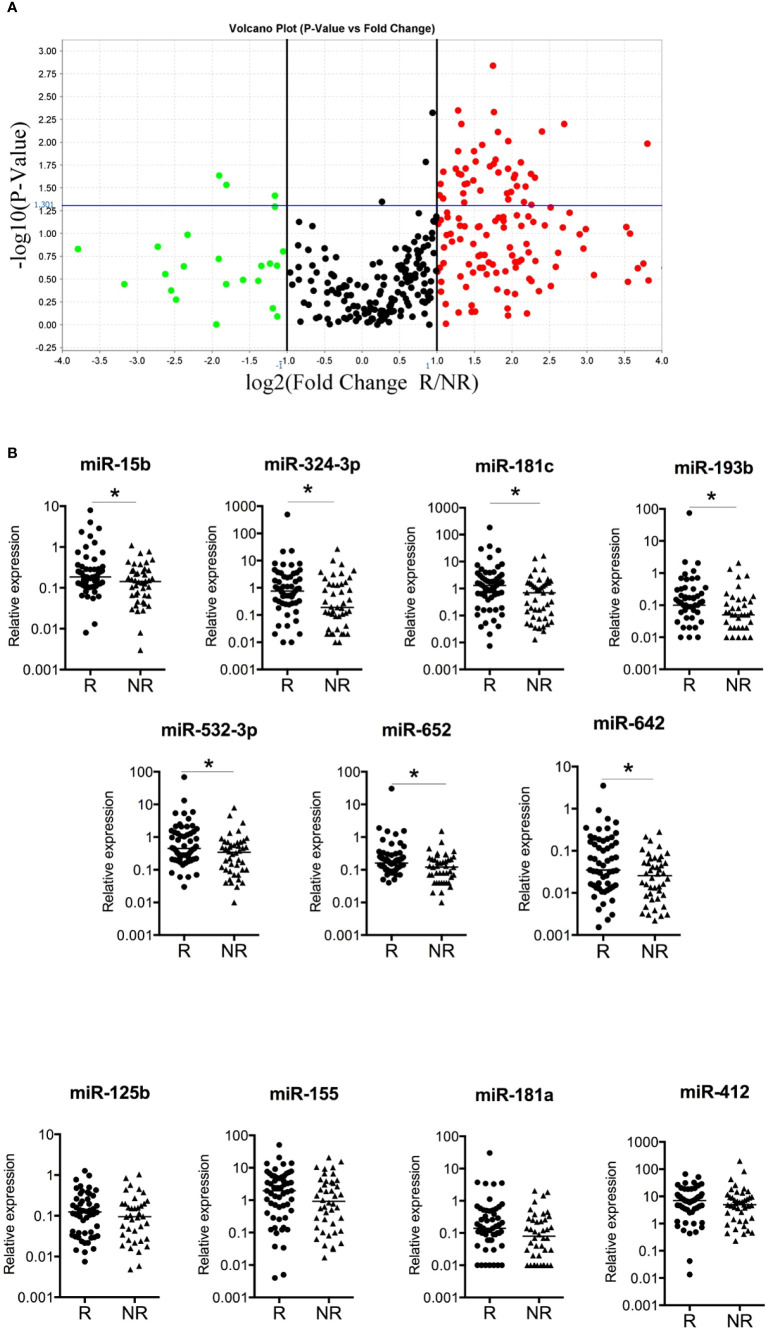
miRNAs associated with complete remission and uMRD **(A)** miRNAs associated with CR and BM uMRD (undetectable minimal residual disease) in previously untreated B-CLL patients. A volcano plot compares the normalized expression of miRNAs in first line of treatment outcome in CR with blood and BM uMRD patients (n=8) versus no-CR with detectable MRD value patients (n=8) assessed three months after treatment. Log2 fold changes and their corresponding P-values of all genes in the microarray were taken for construction of the volcano plot. The y-axis is the negative log10 of P values (a higher value indicates greater significance) and the x-axis is the difference in expression between two experimental groups. Genes up-regulated with more than 2.5-fold change are depicted in red boxes and those downregulated with identical fold change are in green boxes. Genes above the blue line are significantly deregulated (P-value<0.05, t-test). All other genes in the array that were not deregulated are in black dots. 25 genes were identified as significantly up or downregulated. **(B)** The expression level of 11 miRNAs was quantified using multiplex RT-qPCR in a validation cohort of treatment-naïve B-CLL patients (n=100). R: responders, NR: non-responders. Median are plotted, * P-value<0.05.

To identify a blood-based miRNA signature in treatment-naive patients with B-CLL that could predict clinical response, we first used a real-time PCR-based high-throughput miRNA array approach and compared the miRNA expression profiles between 8 B-CLL patients in CR with BM uMRD and 8 B-CLL patients in no-CR with detectable BM MRD. The volcano plot displays the relationship between Log2 fold-change (FC) and significance (P-value<0.05) between both groups for 384 miRNAs screened, using a scatter plot view. A total of 46 miRNAs were differentially expressed (2.5-fold), of which 3 miRNAs were upregulated and 43 miRNAs were downregulated (**Figure 1A**). Among these 46 significantly deregulated miRNAs, only 25 fulfilled acceptable ranges for detection limit (Ct values <30 cycles), over-expression being consistently observed (92% of miRNAs) in patients with CR with BM uMRD as compared with other patients (**Table 2**). To validate miRNA profile by RT-qPCR, we picked 11 out of 25 miRNAs significantly deregulated, representative of the fold change distribution and of their involvement in function and development of B cells and B-CLL (**Figure 1B**).

**Table 2:** List of miRNAs significantly deregulated in responders with BM uMRD versus non responders to first line treatment.

Target Name	Fold Change	p-value
**hsa-miR-412**	0.3	0.023
**hsa-miR-155**	0.3	0.029
**hsa-miR-519a**	2.6	0.019
**hsa-miR-744**	2.6	0.028
**hsa-miR-339-5p**	2.8	0.026
**hsa-miR-652**	2.8	0.013
**hsa-miR-212**	2.9	0.016
**hsa-miR-92a**	3.0	0.011
**hsa-miR-125b**	3.3	0.018
**hsa-miR-15b**	3.4	0.001
**hsa-miR-324-3p**	3.4	0.017
**hsa-miR-193a-5p**	3.4	0.005
**hsa-miR-423-5p**	3.5	0.008
**hsa-miR-139-5p**	3.5	0.021
**hsa-miR-181c**	3.9	0.02
**hsa-miR-193b**	3.9	0.01
**hsa-miR-211**	4.1	0.025
**hsa-miR-328**	4.1	0.023
**mmu-miR-134**	4.2	0.021
**hsa-miR-494**	4.3	0.017
**hsa-miR-532-3p**	4.5	0.031
**hsa-miR-181 a**	4.8	0.022
**hsa-miR-642**	4.9	0.024
**hsa-miR-99b**	5.3	0.008
**hsa-miR-202**	6.5	0.006

Fold-change is the fold-ratio geometric means of miRNA expression in B-CLL patients presenting a CR with BM uMRD versus no-CR with detectable MRD. Red miRNAs were selected for validation on the entire cohort by RTqPCR. CR, Complete response; BM, Bone Marrow; MRD, Minimal residual disease.

### Blood miRNAs predictive of the treatment outcome

Univariate analysis of these 11 miRNAs showed that the overexpression of 4 miRNAs (miR-15b, -324-3p, -532-3p, and -652) before immuno-chemotherapy was associated with the clinical response (CR versus no-CR) in B-CLL patients (**Table 3**). Only miR-125b was statistically predictive of a blood uMRD (p=0.029). Seven miRNAs were predictive of a BM uMRD, and 8 miRNAs were significantly associated with a CR and BM uMRD. In the group of patients with favorable outcome, miRNA expression levels were always higher than in the group of patients with unfavorable outcome (CR vs no-CR patients, blood uMRD vs MRD positive patients, BM uMRD vs MRD positive patients, CR with BM uMRD vs other patients). Multivariate analysis showed that Binet stage AB (5.47 [1.44-35.95]; p=0.011) and a high expression level of miR-15b (4.52 [1.55-23.05]; p=0.003) were significantly associated with a superior likelihood of achieving CR with BM uMRD.

**Table 3:** Univariate miRNA analyses in B-CLL patients according to treatment response using the IWCLL 2008 guidelines and uMRD.

	CR	Blood uMRD	BM uMRD	CR + uMRD
**miR15b**	0.017	ns	0.024	0.004
**miR125b**	ns	0.029	ns	ns
**miR155**	ns	ns	ns	ns
**miR181a**	ns	ns	0.024	0.027
**miR181c**	ns	ns	0.017	0.013
**miR193b**	ns	ns	0.007	0.005
**miR324-3p**	0.024	ns	0.008	0.004
**miR412**	ns	ns	ns	ns
**miR532-3p**	0.018	ns	0.021	0.003
**miR642**	ns	ns	ns	0.030
**miR652**	0.014	ns	0.012	0.002

Two groups of responders were defined by achieving a CR vs no-CR, uMRD vs MRD positive, and CR with uMRD vs no-CR whatever the MRD or CR with detectable MRD. MRD is the evaluation of immunophenotypic response by sensitive 8-color flow cytometry in both blood and bone marrow. Patients with no-CR, with blood MRD positive, with BM MRD positive, or group composed with no-CR patients whatever the MRD or CR patients with detectable MRD are used as the OR reference group. CR, Complete response; uMRD, Undetectable minimal residual disease; BM, Bone marrow.

**Figure 2:**
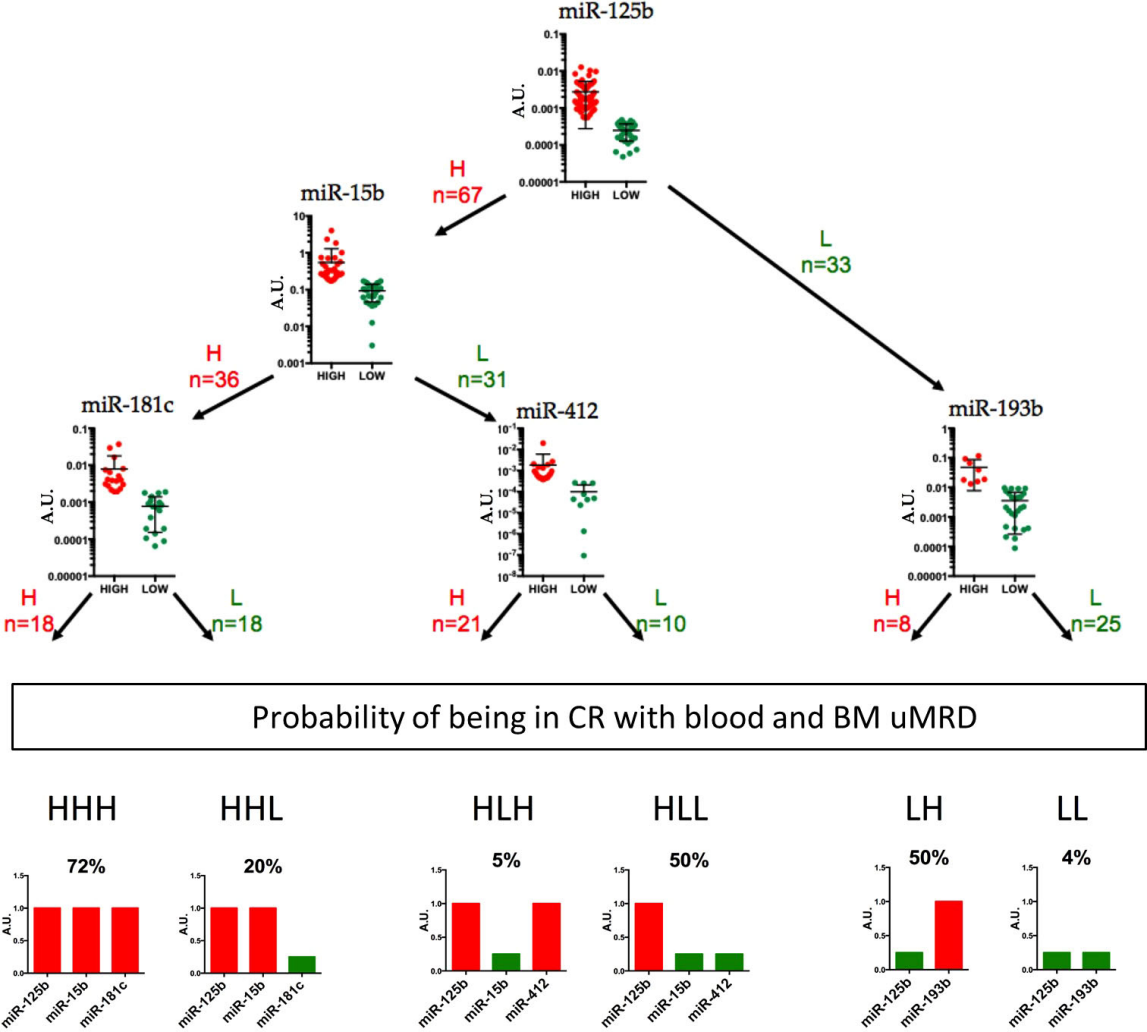
A decision-making model using expression of 5 blood miRNAs at baseline to classify B-CLL patients and predict treatment outcome. Receiver Operating Characteristic (ROC) curves method was used to constructed the decision tree, underlying 6 groups of patients. For each group, relative expression of 2 or 3 miRNAs into the blood of B-CLL patients (n=100) can give the probability at diagnosis of being in CR with BM uMRD three months treatment termination. Red and green colors were used for high (H) and low (L) miRNA expression levels.

Using miRNA expression levels and clinical parameters before treatment, we produced a decision tree to predict by blood sampling the probability to obtain CR with BM uMRD (**Figure 2**) 3 months after FCR treatment. Because patients with both detectable blood MRD and BM uMRD do not exist and because miR-125b was the only miRNA that could statistically predict blood uMRD after treatment, we used the expression level of miR-125b at diagnostic as a root node at the top of the decision tree. Receiver operating characteristic (ROC) curves were constructed to determine the threshold able to predict CR with BM uMRD patients with the best sensitivity and specificity. All parameters of ROC curves used to design the decision tree are detailed in **Table 4**. The resulting cut-off was used to discriminate patients with high and low miR-125b expression levels. For each group of patients (high versus low miRNA expression), univariate statistical analysis was performed. The input variable with the most significant p-value was used to estimate the likely value in the target variable. The decision tree was finalized when no more variable was statistically different between CR with BM uMRD patients and other patients. Six groups of patients were defined depending on 2 to 3 miRNAs expression levels with a distinct probability of being CR with BM uMRD going from 72% (miR-125b high, miR-15b high and miR-181c high) to 4% (miR-125b low and miR-193b low). Of note, *IGHV* mutational status did not influence the classification of CR with BM uMRD patients in the miRNA-based decision tree (Data not shown). Interestingly, blood lymphocytes but not CD20 expression at diagnosis are significantly different between the six groups of patients, high levels of lymphocytes being associated with the LL group (lowest probability of response) (**Supplementary Table 2**).

**Table 4:** Biomarker potential of 5 miRNAs.

	p values	Threshold	Sp (%)	Se (%)
**miR-15b**	0.0064	0.1710	56	76
**miR-125b**	0.0231	0.0005	53	78
**miR-181c**	0.0244	0.0019	71	81
**miR-193b**	0.0420	0.0113	86	80
**miR-412**	0.0365	0.0003	82	80

Threshold and p-values of ROC curves used to evaluate the prognostic potential of the 5 miRNAs used to build the decision tree.

### Progression free survival and miRNAs

**Figure 3:**
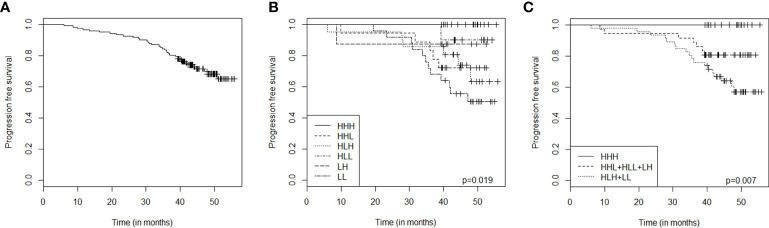
Progression free survival (PFS) in the total cohort according to the five-miRNA decision tree signature. PFS in the total cohort **(A)**, according to the six different groups of signatures identified in the miRNA decision tree **(B)**, or with combined miRNA signature groups **(C)**. PFS, Progression free survival; HHH, group of patients with miR-125b high, miR-15b high and miR-181c high; HHL, group of patients with miR-125b high, miR-15b high and miR-181c low; HLH, group of patients with miR-125b high, miR-15b low and miR-412 high; HLL, group of patients with miR-125b high, miR-15b low and miR-412 low; LH, group of patients with miR-125b low and miR-193b high; LL, group of patients with miR-125b low and miR-193b low; HHL+HLH+HLL+LH, consolidation of HHL-, HLH-, HLL-, and LH-group.

At a median follow-up of 42.4 months (IQR=37.1-47.0), median progression free survival (PFS) and median event free survival (EFS) were not reached. PFS was 71% (**Figure 3**). Univariable associations between baseline characteristics and PFS are shown in **Table 5A**. At a median time of 42.4 months post-treatment, PFS was 84% for patients with *IGHV*-mutated and 64% for patients with *IGHV*-unmutated (p=0.012) (data not shown). Nine months landmark PFS was performed according to *IGHV* mutational status and achievement of post-treatment CR with BM uMRD (p<0.0001). Whatever was their *IGHV* mutational status, none of the BM uMRD patients relapsed after 42.4 months of follow-up. In detectable BM MRD patients, PFS was 69% for patients with *IGHV*-mutated and 47% for patients with *IGHV*-unmutated (data not shown).

**Table 5:** Univariate analysis of PFS according to baseline characteristics and miRNAs.

A	HR (95% CI)	p value
**Age ≥58.5, n=61**	1.02 (0.53-1.95)	0.965
**Male Gender, n=90**	1.05 (0.49-2.24)	0.895
**Binet stage AB, n=91**	0.80 (0.39-1.62)	0.534
**ECOG 1, n=37**	1.68 (0.89-3.28)	0.126
**Unmutated IGHV, n=75**	2.78 (1.21-6.41)	0.012
**Cytogenetic abnormalities**		
**Del(13q), n=54**	0.61 (0.29-1.28)	0.188
**Del(11q), n=24**	1.79 (0.84-3.84)	0.128
Trisomy 12, n=9*	–	0.039
**Lymphocyte count >71(G/L), n=61**	2.70 (1.32-5.49)	0.004
**β2 microglobulin >2 (mg/L), n=101**	2.46 (0.59-10.25)	0.202
**Treatment arm FCR, n=62**	1.09 (0.57-2.09)	0.802
**B**		
**miR-15b high, n=58**	1.02 (0.53-1.97)	0.948
**miR-125b high, n=67**	0.41 (0.19-0.89)	0.021
**miR-181c high, n=29**	0.49 (0.19-1.26)	0.131
**miR-193b high, n=40**	0.50 (0.22- 1.14)	0.093
**miR-412 high, n=84**	1.12 (0.55-2.29)	0.754

Hazard ratios for PFS according to baseline characteristics (A) and miRNAs (B). *Indicates that 9 data are not available. No progression or progression event were registered in trisomy 12 group. The thresholds of the 5 miRNAs used to build the decision tree are presented. PFS, Progression free survival; HR, Hazard ratio; CI, Confidence interval.

Univariable associations between miRNA expression levels measured before treatment and PFS are shown in **Table 5B**. MiRNAs’ thresholds used to build the decision tree were chosen. Using a multivariate analysis that combines baseline characteristics and the 5 miRNAs of the decision tree, only *IGHV*-unmutated (hazard ratio (HR)=2.41 [1.04-5.58]; p=0.041) and lymphocyte count >71G/L (HR=2.47 [1.17-5.22]; p=0.018) were significantly associated with inferior PFS. Depending on the decision tree group, PFS was significantly different (p=0.019) (**Figure 2**). None of the patients displaying high expression levels of miR-125b, miR-15b and miR-181c (HHH group) relapsed during the follow-up. In contrast, patients with miR-125b high, miR-15b low and miR-412 high (HLH group), and patients with low expression levels of miR-125b and miR-193b (LL group) relapsed more frequently than any other patients, and their 42.4 months PFS were 71% and 52%, respectively. For the four other groups: patients with miR-125b high, miR-15b high and miR-181c low (HHL group), patients with miR-125b high, miR-15b low and miR-412 low (HLL group) and patients with miR-125b low and miR-193b high (LH group), 42.4 months PFS were 72%, 90%, and 87% respectively. PFS curves were constructed by combining these three groups (**Figure 3C**).

### High-risk patients from HLH and LL groups are characterized by enrichment of genes related to genomic instability and mRNA metabolism

**Figure 4:**
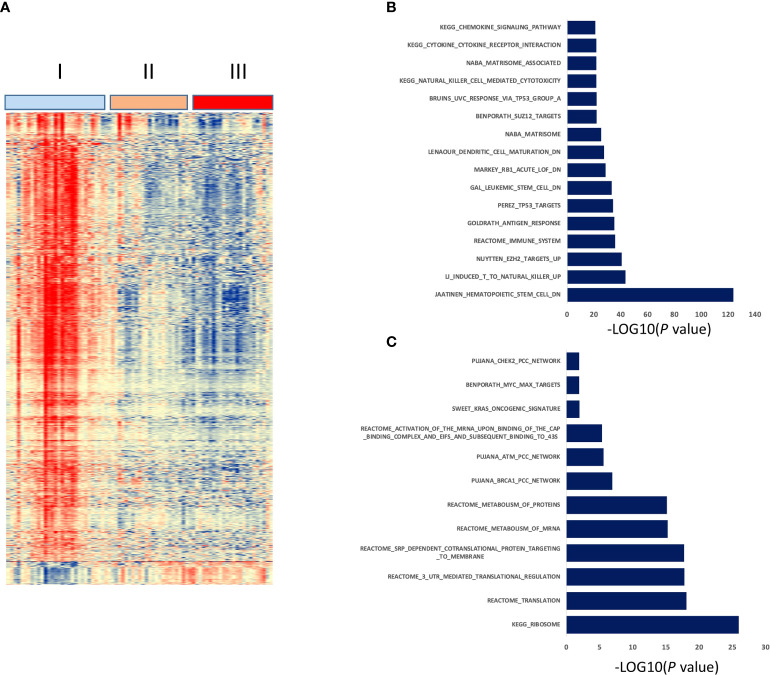
Gene expression profiling of three prognostic groups defined by the five-miRNA decision tree signature. Clustergram of the 1373 differentially expressed genes between HHH group and HLH and LL groups (FDR < 0.05). Signals are displayed from low (deep blue) to high (deep red) expression **(A)**. Molecular signatures enriched in HHH group **(B)** or HLH and LL groups **(C)** using GSEA Database (all curated gene sets), and relevant pathways are presented (FDR q value ≤ 0.05).

To further investigate the molecular mechanisms, we performed RNA-sequencing of blood samples before treatment and compared gene expression profiling between high-risk patients (classified in the HLH and LL groups) and the HHH group characterized by no relapse (**Figure 4**). We identified 1373 DEG between both groups of patients (FDR < 0.05), with 67 genes overexpressed in samples of the HLH and LL groups and 1309 overexpressed in the HHH group (**Figure 4A**). Gene set enrichment analysis (GSEA) analysis demonstrated a significant enrichment of genes related to genomic instability and mRNA metabolism in the high risk HLH and LL groups whereas HHH group is delineated by a significant enrichment in genes related to immune response, interaction with the microenvironment, TP53/RB1 targets and downregulation of polycomb PRC2 target genes (**Figure 4B, C**).

## Discussion

Despite progress in developing chemotherapy-free treatments for B-CLL patients, powerful tools predicting at diagnosis those who will achieve a progression-free survival are still missing. In the present study, we investigated the blood of untreated B-CLL patients to identify a miRNA-based signature that predicts patients who will present a CR with BM uMRD 3 months after therapy and characterized by no relapse. Taking advantage of an existing cohort of 123 patients treated by FCR immuno-chemotherapy (25), we identified a signature of 25 miRNAs associated with CR and BM uMDR, which we used to produce a 5 miRNA-based decision tree to predict by simple blood sampling the probability to obtain (or not) CR with BM uMRD. Our data also confirm that B-CLL patients display abnormal circulating levels of miRNAs and reinforce the critical role of miRNAs in B-CLL pathogenesis.

Among the 25 miRNAs differentially expressed before treatment in the blood of responders with BM uMRD compared with non-responders (**Table 2**), thirteen have been previous reported abnormally expressed in B-CLL and others are described in AML cases. The majority of these miRNAs increased in FCR responders are reduced in PBMC or B cells purified from CLL patients as compared to healthy donors, display tumor suppressor functions and are proposed as predictive biomarkers of prolonged overall survival (30–33). Although the oncogenic miR-17∼92 cluster is frequently upregulated in malignancies including B-CLL (34, 35), miR-92a was the only miRNA deriving from the miR-17∼92 cluster present in our 25-miRNA signature. Of note, its expression was 3-fold higher in the blood of B-CLL patients presenting a CR with BM uMRD than in B-CLL patients with no-CR and detectable MRD. This is in agreement with previous work from Papageorgiou et al. who found that miR-92a-3p is overexpressed in normal PBMC and represents a potential surrogate biomarker of favorable outcome of CLL patients (33), and with the work of Olive et al. who evidenced that co-transcription does not necessarily means cooperation as miR-92a counteracts the effects of miR-19 during the growth and development of B-cell lymphomas (36). Overall, these findings tend to suggest that patients who will respond to FCR treatment would have a circulating level of those miRNAs closer to healthy donors than the non-responders.

Here, we showed that the quantification of miR-15b, miR-125b, miR-181c, miR-193b and miR-412 in the blood of B-CLL patients is sufficient to classify them in 6 groups with a specific probability of response rate for FCR treatment outcome. The probability to obtain CR with BM uMRD for each group goes from 72% when blood levels of miR-125b, miR-15b and miR-181c are high (HHH group) to 4% when blood levels of miR-125b and miR-193b are low (LL group). Moreover, by observing PFS for each of those 6 groups, we found that none of patients relapse in the best predicted treatment outcome group (HHH group). In contrast, the two worse predicted treatment outcome groups (HLH and LL groups) had the lower PFS. Intermediate PFS curves correlate with intermediate groups of responders (HHL, HLL and LH groups). Based on these observations, we established three statistically different PFS groups: high PFS (HHH group), intermediate PFS (HHL, HLL and LH groups) and low PFS (HLH and LL groups). Interestingly, as blood lymphocytosis (but not with CD20 expression levels) at diagnosis is significantly associated with the 6 different groups of predicted responders, the expression level of some if not all 5 miRNAs used for the decision tree might be associated with non-CLL cells.

Comparing gene expression profiling between high (HHH group) and low PFS (HLH and LL groups) groups, we identified that high risk group is characterized by enrichment of genes related to genomic instability and mRNA metabolism. Of note, a ribosome-related signature was reported to be associated with poor outcome in CLL (37). Patients with a sustained PFS presented enrichment in gene signatures related to immune response, interaction with the microenvironment, TP53/RB1 targets and downregulation of polycomb PRC2 target genes. Interestingly, EZH2 (the catalytic subunit of PRC2 complex) enhances tumorigenesis and is overexpressed in poor-prognostic CLL patients in association with upregulation of PI3K/AKT through IGFR1 and MYC (38) and epigenetically silences the tumor suppressor miR-125 (39). This is consistent with our observation of reduced expression of EZH2 and increased expression of miR-125 in the best prognostic HHH group of patients. Our data are in agreement with the essential role played by tumor microenvironment in the development, growth and survival of the malignant B-cell clone in CLL. Since the immunosuppressive state of the tumor microenvironment in CLL (40) is delineated by exhausted effector T cells, our results suggest that the HHH group may be characterized by more efficient immune mircroenvironment. Indeed, GSEA of the HHH group of patients revealed an upregulation of genes associated with an antigen response of CD8+ T cells, with a NK cell-mediated cytotoxicity, and with dendritic cell differentiation and maturation. Based on this, one could suggest that the HHH group of patients may be better armed to control leukemia thanks to an active anti-CLL immunity involving CD8+ T cells, NK and dendritic cells.

When searching for validated miRNA–mRNA target interactions that could be functional (41), we identified 2 genes encoding for ribosomal proteins RPS3A and RPS7 as putative target mRNAs for miR-125b and miR-15b among all genes that are downregulated in the HHH group of patients. RPS3A and RPS7 are involved in nuclear-transcribed mRNA catabolic processes including nonsense-mediated decay. Interestingly, high level of RPS3A correlates with low immune cell infiltration in hepatic tumor and bad prognosis (42), and a ribosome-related signature in CLL blood is associated with reduced survival (37). This is in agreement with our findings that high levels of miR-125b and miR-15b are associated with good prognosis for B-CLL patients. Investigation about the role of miRNAs on the RPs and consequences on the biogenesis of ribosomes and global gene expressions are limited. However, we speculate that, by repressing the expression of RPS3A and RPS7 ribosomal proteins, miR-125b and miR-15b might alleviate the breakdown of aberrant RNAs, altering the translatome of B-CLL cells in a wat that results in acquisition of a less aggressive resting phenotype.

Our results are independent of the *IGHV* mutational status or cytogenetic abnormalities. Results of long-term remissions after FCR chemoimmunotherapy in previously untreated patients with B-CLL confirm the importance of achieving uMRD, in particular for *IGHV*-mutated patients (14). The repartition of patients in CR with BM uMRD in our three groups of PFS (from better to the worse outcome: 18%, 50% and 25%) was approximately equivalent to those in the Thompson study (21% for *IGHV*-mutated/uMRD patients, 41% for regrouped *IGHV*-mutated/MRD-positivity and *IGHV*-unmutated/uMRD patients, and 39% for *IGHV*-unmutated/MRD-positivity patients). Importantly, Thompson’s results were obtained after the end of the treatment while our miRNA sampling is performed at diagnosis. Bone marrow biopsy is not used as a matter of routine, but only in clinical trial to confirm the effectiveness of the treatment by the absence of residual disease. Peripheral blood MRD is not sensitive enough and not as efficient as BM MRD. Being able to predict response before treatment based on a non-invasive blood test as simple and easy as a 5-miRNAs multiplex RT-qPCR is a powerful tool for clinicians to stratify patients and optimize the medical treatment decision. It is indeed important to avoid exposing patients to chemotherapy-associated toxicities if their probability of being in CR with BM uMRD is less than 15%.

The five miRNAs found implicated in the FCR treatment outcome have been previously described in cancers, leukemias and B-CLL. The primary transcripts (pri-miRNAs) of miR-15a/-16/-15b are elevated and their processing into precursor miRNAs is reduced in cells from B-CLL patients compared to non-malignant B-cells (43). These data indicate a blockade of miRNA biogenesis at the DROSHA processing step and might thus suggest that all miRNA biogenesis is reduced in B-CLL, indirectly implying that miRNA biogenesis might be preserved in cells of good responders to FCR treatment (HHH group). In addition, the miR-15/miR-16 cluster is located in a region that is deleted in more than half of B-CLL cases (44). Finally, the tumor suppressor function of this cluster in CLL was demonstrated by the increased development of B-cell malignancies in the miR-15b/16-2 knockout mouse (45), which is in line with the control by miR15b/16-2 of the CCND2 (Cyclin D2), CCND1 and IGF1R (insulin-like growth factor 1 receptor) genes involved in proliferation and antiapoptotic pathways in mouse B cells. Moreover, the expression of miR-125b and miR-181c in B-CLL purified B cells is downregulated in comparison with healthy donors (46, 47), which is consistent with the negative regulation of the *TCL1* oncogene by miR-181 B-CLL (48) and of several transcripts involved in cell metabolism by miR-125b (47), and with the observation that miR-125b up-regulation is associated with aggressive leukemia in human and mice (49, 50). The implication of miR-193b in leukemias, such as AML (51), T-cell lymphoblastic lymphoma (52, 53) or splenic marginal zone lymphoma (54) has been shown. According to the subtype of leukemia, miR-193b can be inversely correlated with c-kit levels, linked to cell growth (51), contributing to the expression of Smoothened to activate the GLI/Hh signaling, promoting cell survival and proliferation (52), or targeted the MYB oncogene, MCL1, CCND1, 14-3-3, SHMT2, AKR1C2 (53, 54). This miRNA is also downregulated in B-CLL cells from early-stage cases compared to B memory normal cells (32). Studies on miR-412 are however scarce. Single–nucleotide polymorphisms of pri-miR-409_miR-412 (rs61992670) and mat-miR-412 (rs61992671) are detected in B-CLL patients and the allelic frequency of mat-miR-412 was significantly different between B-CLL patients and 1000 genomes project samples (55). Finally, the miR-15/miR-16 cluster, miR-125b, miR-181 family members and miR-193b, which display high levels at diagnosis in B-CLL patients who will present a CR with BM uMRD after FCR treatment, are key regulators of normal hematopoiesis and of immune cell functions. All 4 miRNAs are also downregulated in a RNAseq study of B cells purified from CLL patients as compared to healthy donors (31).

Further investigations are needed to investigate the impact miRNA-based signature in patients treated with BTKi or BCL2i, which are now the treatment of B-CLL patients allowing to have early and easy assessable markers predicting outcome of patients.

## Data availability statement

The data presented in the study are deposited in the Gene Expression Omnibus repository, accession number GSE214763. https://www.ncbi.nlm.nih.gov/geo/query/acc.cgi?acc=GSE214763.

## Ethics statement

The studies involving human participants were reviewed and approved by the CPP Sud méditerranée IV. The patients/participants provided their written informed consent to participate in this study. No animal studies are presented in this manuscript.

## Author contributions

Authors contributions were: Design of research (GC, FA, ID-R), Performing research (A-LG, GC, FA, ID-R, OK, RL, JM, EA, ET), Data collection (GC, RL, SL, JM, EA, ID-R, OK, ET), Data analyses and interpretion (A-LG, GC, FA, ID-R, JM, EA), Performed statistical analyses (A-LG, ID-R), Writting & reviewing the manuscript (A-LG, GC, FA, ID-R, JM, RL). Finally, all authors approved the submitted version.

## Funding

The French Innovative Leukemia Organization (FILO) group funded the clinical trial that served for this experimental work. FILO was the sponsor and was involved in managing the clinical study, statistical analyses, and data review. Roche provided rituximab (MabThera) but had no role in study design or data collection, analysis, or interpretation. The authors declare that experimental work presented in this article received fundings from a public grant overseen by the French National Research Agency (ANR) ANR-18-CE15-0010-01 and as part of the “Investissements d’Avenir” program (reference Labex MAbImprove: ANR-10-LABX-53-01), INSERM (Institut National de la Santé et Recherche Médicale), the University of Montpellier, the Institut Universitaire de France, the “Groupement Interrégional de Recherche Clinique et d’Innovation Sud-Ouest Outre-Mer Hospitalier” (APIK 2017) and the “Cancéropôle Grand-Sud-Ouest”. These later funders were not involved in the study design, collection, analysis, interpretation of data, the writing of this article or the decision to submit it for publication.

## Acknowledgments

We would like to dedicate this publication to the memory of Isabelle Duroux-Richard who was a driving force behind this work and sadly passed away suddenly this year.

## Conflict of interest

Author GC received consultancy by Roche and BMS and honoraria from Roche, BMS, Jansen, Novartis and Gilead. Author ET received consultancy and honoraria from Janssen and Abbvie. Author SL received honoraria from Gilead, Janssen, Beigene, Abbvie, Astra Zeenca. Author OK was employed by Erasmus Mundus (2013-2016).

The remaining authors declare that the research was conducted in the absence of any commercial or financial relationships that could be constructed as a potential conflict of interest.

## Publisher’s note

All claims expressed in this article are solely those of the authors and do not necessarily represent those of their affiliated organizations, or those of the publisher, the editors and the reviewers. Any product that may be evaluated in this article, or claim that may be made by its manufacturer, is not guaranteed or endorsed by the publisher.

## Supplementary material

Supplementary Table 1 Association between patients’ characteristics and achievement of CR, uMRD and CR with BM uMRD. CR, Complete remission; MRD, Minimum Residual Disease; uMRD, undetectable Minimum Residual Disease BM, Bone Marrow.; OR, Odds ratio; CI, Confidence Interval; Ind, indeterminable. Patients with no-CR, with MRD-positive or with no-CR-uMRD in BM are used as the OR reference group.

Supplementary Table 2 Association between blood lymphocytosis at diagnosis and the six miRNA-based prognostic signatures. Mediane values of blood lymphocyte counts (G/L) and CD20 expression (MESF: molecules of equivalent soluble fluorochrome) at diagnosis are given for the six groups of B-CLL patients (n=100) classified into different probabilities of responding to three months of FCR treatment according to the high (H) versus low (L) expression level of 5 miRNAs.
