# Supplementary material for: miRNA profile at diagnosis predicts treatment outcome in patients with B-chronic lymphocytic leukemia: A FILO study
Source: Front Immunol. 2022 Oct 17;13:983771. doi: 10.3389/fimmu.2022.983771 (PMC9618812; doi:10.3389/fimmu.2022.983771)
Supplement: Supplementary Table 1 — Association between patients’ characteristics and achievement of CR, uMRD and CR with BM uMRD. CR, Complete remission; MRD, Minimum Residual Disease; uMRD, undetectable Minimum Residual Disease BM, Bone Marrow.; OR, Odds ratio; CI, Confidence Interval; Ind, indeterminable. Patients with no-CR, with MRD-positive or with no-CR-uMRD in BM are used as the OR reference group. [file Table_1.docx]

# Supplementary Tables

# TABLE S1 Association between patients’ characteristics and achievement of CR, uMRD and CR with BM uMRD

CR, Complete remission; MRD, Minimum Residual Disease; uMRD, undetectable Minimum Residual Disease BM, Bone Marrow.; OR, Odds ratio; CI, Confidence Interval; Ind, indeterminable. Patients with no-CR, with MRD-positive or with no-CR-uMRD in BM are used as the OR reference group.

TABLE S2 Association between blood lymphocytosis at diagnosis and the six miRNA-based prognostic signatures

|  | **HHH** | **HHL** | **HLH** | **HLL** | **LH** | **LL** | **p value** |
| --- | --- | --- | --- | --- | --- | --- | --- |
| **Lymphocytes (G/L)** | 11.4 | 56.3 | 71 | 50.2 | 96.2 | 109 | <0.001 |
| **CD20 (MESF)** | 16 607 | 9 630 | 10 294 | 11 618 | 7 503 | 11 193 | 0.412 |

Mediane values of blood lymphocyte counts (G/L) and CD20 expression (MESF: molecules of equivalent soluble fluorochrome) at diagnosis are given for the six groups of B-CLL patients (n=100) classified into different probabilities of responding to three months of FCR treatment according to the high (H) versus low (L) expression level of 5 miRNAs.
